# Characteristics of Opioid-Dependent Patients in the Inpatient Setting: A Cross-Sectional Study in Riyadh, Saudi Arabia

**DOI:** 10.7759/cureus.111838

**Published:** 2026-06-30

**Authors:** Meshal M Zuraie, Hassan Khubrani, Faisal Alzkkri, Marwa Abdelmoaty, Saad Alqahtani, Mohamed Abdelhalim Darweesh

**Affiliations:** 1 Mental Health, King Abdulaziz Medical City, Riyadh, SAU; 2 Addiction Services, Eradah Mental Health Complex, Riyadh, SAU; 3 Psychiatry, King Khalid University Hospital, Riyadh, SAU; 4 Psychiatry, Eradah Mental Health Complex, Riyadh, SAU; 5 Addiction Services, Armed Forces Hospital Southern Region, Khamis Mushait, SAU

**Keywords:** buprenorphine, heroin injection complication, inpatient addiction treatment, opioid use disorder, polysubstance use

## Abstract

Background: Opioid use disorder (OUD) remains a significant public health concern in Saudi Arabia; however, recent evidence describing patient characteristics and treatment outcomes in inpatient settings is limited. The objective of this study was to describe the demographic, clinical, and treatment profiles of inpatients with OUD and assess early post-discharge outcomes, following buprenorphine-based therapy.

Methods: This descriptive cross-sectional chart review was conducted at the Eradah Mental Health Complex in Riyadh, Saudi Arabia. Medical records of 22 male inpatients admitted between April and September 2024 with confirmed OUD alone or in combination with other substances were reviewed. Extracted data included socio-demographics, substance use history, comorbidities, withdrawal symptoms, treatment regimens, prevalence of polysubstance use, treatment types, discharge status, and six-month relapse or readmission rates.

Results: The mean age of patients was 38.3 years. Most were Saudi nationals (n=13, 59.1%) and unemployed (n=19, 86.4%). Heroin was the most commonly used opioid (n=14, 63.6%). Polysubstance use was highly prevalent (n=20, 90.9%), with methamphetamine/amphetamine reported in 45.5% of patients (n=10). Withdrawal symptoms occurred in 68.2% of patients (n=15), with the most common being insomnia (n=10, 66.7%), muscle aches (n=8, 53.3%), and sweating (n=6, 46.7%). Medical comorbidities were documented in 45.5% (n=10). Buprenorphine/naloxone induction followed by transition to weekly long-acting injectable buprenorphine was the most common regimen (n=12, 54.5%). Planned discharges were documented in 63.6% of patients (n=14), while six-month readmissions occurred in 27.3% (n=6). Loss to follow-up was recorded in 22.7% (n=5).

Conclusion: This inpatient cohort showed frequent polysubstance use, medical comorbidity, non-employment, and prior treatment exposure. Buprenorphine-based opioid agonist therapy (OAT) was commonly used, including long-acting injectable formulations, but observed associations with discharge pathways are preliminary and should not be interpreted as causal or as evidence of treatment effectiveness. Larger multicenter studies with standardized follow-up are needed.

## Introduction

Opioid use disorder (OUD) is a chronic relapsing condition characterized by impaired control over opioid use, craving, tolerance, withdrawal, and continued use despite clinically significant harm [1,2]. Although opioids have an established role in pain management, prolonged unsupervised use, non-medical use, or misuse can lead to dependence, overdose, and substantial morbidity [2].

Globally, opioids contribute substantially to drug-related morbidity and mortality. The World Health Organization (WHO) reported that approximately 60 million people used opioids worldwide in 2021 and that 39.5 million people lived with drug use disorders. In 2019, approximately 600,000 deaths were attributable to drug use, close to 80% were related to opioids, and approximately 125,000 deaths were due to opioid overdose [3].

Polysubstance use and comorbidity are central challenges in OUD care. Concurrent non-opioid substance use may complicate withdrawal management, reduce treatment engagement, and increase relapse risk. A recent systematic review and meta-analysis found that current non-opioid substance use disorders were common among people with OUD, most frequently involving cocaine, alcohol, cannabis, sedatives, and methamphetamine [4]. Medical and psychiatric comorbidities further increase the complexity of treatment planning and post-discharge follow-up [2,4].

In Saudi Arabia, evidence on opioid use and OUD remains limited, particularly in inpatient settings. Available local data are derived mainly from retrospective, hospital-based, and treatment-center studies rather than population-level epidemiological surveys. Earlier Saudi studies have described heroin, amphetamines, alcohol, cannabis, and polysubstance use among selected treatment-seeking populations; however, these findings vary by setting, study period, and sample characteristics and should not be interpreted as national prevalence estimates or population trends [5-9].

Medical comorbidity is also clinically relevant, especially among patients with opioid injection use. A retrospective study of male Saudi inpatient heroin users treated at a primary specialized addiction treatment center serving the central region of Saudi Arabia reported hepatitis C virus (HCV) antibody seropositivity in 77.8%, human immunodeficiency virus (HIV) infection in 9.8%, and hepatitis B virus (HBV) surface antigen positivity in 7.7% of the participants [10].

Opioid agonist therapy (OAT), including buprenorphine-based treatment, is an established pharmacological option for OUD. Sublingual buprenorphine/naloxone and long-acting injectable buprenorphine may support withdrawal management, craving reduction, and continuity of care when combined with psychosocial and follow-up services [11-13]. However, real-world data on the characteristics and early outcomes of patients receiving buprenorphine-based OAT in Saudi inpatient addiction settings remain scarce. Therefore, this study aimed to describe the demographic, clinical, substance-use, treatment, discharge, and early follow-up characteristics of male inpatients with confirmed OUD treated at a specialized addiction facility in Riyadh, Saudi Arabia.

## Materials and methods

This was a descriptive cross-sectional study that used retrospective data from medical records for patients diagnosed with OUD, either as the sole diagnosis or with another substance use disorder, between April and September 2024. The study was conducted at the Department of Addiction, Eradah Mental Health Complex, Riyadh, Saudi Arabia, a major specialized addiction treatment center serving the central region of Saudi Arabia. The study was approved by the Institutional Review Board of King Fahad Medical City (log number: 24-384E, dated July 18, 2024). The study was conducted in accordance with the Declaration of Helsinki and institutional requirements for retrospective chart review.

Study population

All eligible admissions during the study interval were screened. Although the initial target sample was at least 30 patients, 22 patients met the eligibility criteria and were included in the final analysis. Patients were eligible if they were 18 years of age or older, admitted during the study period, and had a confirmed diagnosis of OUD alone or with another substance use disorder. Confirmation was based on documentation of opioid use and a positive opioid urine toxicology screen before or during admission. Patients were excluded when the admission was for a substance use disorder without current or prior documentation supporting opioid use. It needs to be noted that there were no sex-related restrictions on eligibility; however, all patients who met the study criteria during the study period were male. Therefore, the final sample included only male patients.

Data collection

A structured chart-review form was used to extract variables aligned with the study objectives. These included demographic characteristics, admission pathway, opioid and other substance-use profile, psychiatric and medical comorbidity, selected laboratory and toxicology results, treatment received, discharge outcome, readmission, loss to follow-up, and available urine toxicology results within six months after discharge.

Variables that were inconsistently recorded, including standardized withdrawal scores, craving ratings, adverse effects, medication satisfaction, and detailed tolerability ratings, were not included in the final analysis.

Definitions

OUD was defined according to Diagnostic and Statistical Manual of Mental Disorders, Fifth Edition (DSM-5) criteria, which require at least two diagnostic features such as hazardous use, tolerance, withdrawal, craving, or related impairment [1]. Vital signs were categorized using established clinical references for blood pressure, temperature, pulse, and respiratory rate [14-17]. Body mass index (BMI) categories followed WHO classifications [18].

Readmission within six months was recorded when a patient had a documented inpatient readmission after discharge. Possible relapses were assessed using readmission documentation, clinician notes, and available urine toxicology results; however, toxicology testing was not available for all patients during follow-up.

Statistical analysis

Data were analyzed using IBM SPSS Statistics for Windows, version 23 (IBM Corp., Armonk, New York, United States). Continuous variables are presented as mean and standard deviation (SD), and categorical variables as frequencies and percentages. Fisher's exact test or the chi-square test was used for exploratory comparisons between treatment type and discharge or follow-up outcomes. Because of the small sample size and sparse cell counts, p-values were interpreted descriptively, and no causal or effectiveness conclusions were drawn.

## Results

Participant characteristics

The analysis included 22 male inpatients with confirmed OUD. Thirteen patients (59.1%) were Saudi nationals, and 14 (63.6%) were 25-44 years of age. High school or secondary school was the most frequent educational level, documented in 10 patients (45.5%). Non-employment was documented in 19 patients (86.4%); 14 (63.6%) were single, and legal issues were documented in 10 (45.5%) (Table 1). The current admission lasted a mean of 11.77 ± 4.7 days. The mean weight was 73.22 ± 14.9 kg, the mean height was 168.55 ± 5.8 cm, and the mean BMI was 25.66 ± 4.5 kg/m² (Table 2).

**Table 1:** Demographic characteristics of study participants (N=22)

Characteristics	Category	Frequency	Percentage
Age group (years)	18–24	2	9.1
	25–34	7	31.8
	35–44	7	31.8
	45–54	4	18.2
	55–64	2	9.1
Biological sex	Male	22	100.0
Nationality	Saudi	13	59.1
	Non-Saudi	9	40.9
Highest educational level	Elementary school	3	13.6
	Middle school	4	18.2
	High school / secondary school	10	45.5
	University	4	18.2
	Not reported	1	4.5
Occupational status	Employed	2	9.1
	Non-employed	19	86.4
	Not reported	1	4.5
Marital status	Married	6	27.3
	Divorced	2	9.1
	Single	14	63.6
Legal issues	Yes	10	45.5
	No	9	40.9
	Not reported	3	13.6

**Table 2:** Length of stay and anthropometric measurements upon admission (N=22)

Variable	Frequency	Minimum	Maximum	Mean	SD
Duration of hospital stay (Days)	22	3	21	11.77	4.7
Weight (kg)	22	50	109	73.22	14.9
Height (cm)	22	160	179	168.55	5.8
BMI (kg/m²)	22	19.53	36.20	25.66	4.5

Referrals most often came from the emergency department (ED), accounting for 14 admissions (63.6%). Ten patients (45.5%) presented alone, and nine (40.9%) were accompanied by a family member, relative, or friend. The most common admission reasons were stabilization (n=11, 50.0%) followed by diagnostic/treatment initiation and stabilization (n=10; 45.5% each) (Table 3).

**Table 3:** Admission pathway details (N=22) OPD: outpatient department; ED: emergency department

Current Admission Details	Category	Frequency	Percentage
Source of referral	OPD	8	36.4
	ED	14	63.6
Presented with/Brought by	Family, relative, or friend	9	40.9
	Drug control	1	4.5
	Alone	10	45.5
	Not reported	2	9.1
Reason for admission	Establish diagnosis and treatment	1	4.5
	Stabilize the condition	11	50.0
	All of the above two	10	45.5
Safety concern on admission	Yes	1	4.5
	No	21	95.5

Opioid and other substance use

Heroin was the most frequently documented opioid, reported in 14 patients (63.6%), followed by morphine sulfate in six (27.3%). Opioid use for at least six months was documented in 21 patients (95.5%), and use within 24 hours before admission was documented in 13 (59.1%). Polysubstance use was present in 20 patients (90.9%). The most frequently documented non-opioid substances used with opioids were methamphetamine/amphetamine in 10 patients (45.5%) and cannabis in six (27.3%) (Table 4).

**Table 4:** Opioid and polysubstance use (N=22)

Substance Use Profile	Category	Frquency	Percentage
Opioid type	Heroin	14	63.6
	Morphine sulfate	6	27.3
	More than one opioid type	1	4.5
	No clear documentation, but laboratory result was positive	1	4.5
Opioid method of use	Intravenous	6	27.3
	Snorting or sniffing	1	4.5
	More than one method	2	9.1
	Not reported / not applicable	13	59.1
Use with peers	With peers	4	18.2
	Not reported / not applicable	18	81.8
Duration of use	6 months and longer	21	95.5
	Not reported	1	4.5
Time since last opioid use	Within last 24 hours	13	59.1
	Within last 72 hours	3	13.6
	Within last week	2	9.1
	Not reported	4	18.2
Smoking status	Yes	4	18.2
	No	1	4.5
	Not reported / not applicable	17	77.3
Concurrent substance use	Only opioid	1	4.5
	Polysubstance use	20	90.9
	Not reported	1	4.5
Substance mainly used with opioid among polysubstance users	Cannabis	6	27.3
	Lyrica	1	4.5
	Benzodiazepine	1	4.5
	Methamphetamine/amphetamine	10	45.5
	Not reported / not applicable	4	18.2

Clinical presentation and comorbidities

Withdrawal symptoms were documented in 15 patients (68.2%), absent in five (22.7%), and not reported in two (9.1%). Among patients with documented withdrawal symptoms, the most common symptoms were disturbed sleep or insomnia (n=10, 66.7%), muscle or body aches (n=8, 53.3%), and sweating (n=6, 46.7%). Two patients (9.1%) required high-dependency unit (HDU), medical care unit (MCU), or intensive care unit (ICU) support during admission (Table 5). BMI was categorized as normal in 12 patients (54.5%), pre-obese in six (27.3%), obesity class I in two (9.1%), and obesity class II in two (9.1%)

**Table 5:** Withdrawal symptoms, vital signs, and BMI stratification upon admission (N=22) Note: Multiple responses were permitted. HDU: high-dependency unit; MCU: medical care unit; ICU: intensive care unit

Documented withdrawal symptoms, vital signs, and BMI upon admission	Category	Frequency	Percentage
Required HDU/MCU or ICU care during current admission	Yes	2	9.1
	No	20	90.9
Withdrawal symptoms documented	Yes	15	68.2
	No	5	22.7
	Not reported	2	9.1
Withdrawal symptoms documented	Irritability or anxiety	5	33.3
	Disturbed sleep/insomnia	10	66.7
	Poor appetite	1	6.7
	Generalized fatigability	1	6.7
	Headache	2	13.3
	Muscle pain/body aches	8	53.3
	Abdominal pain or cramps	4	26.7
	Vomiting	2	13.3
	Nausea	2	13.3
	Diarrhea	2	13.3
	Lacrimation/tearing	4	26.7
	Runny nose	5	33.3
	Sweating	7	46.7
Temperature	35.0–36.4°C	6	27.3
	36.5–37.3°C, normal	16	72.7
Systolic blood pressure	<120 mmHg	5	22.7
	120–129 mmHg	10	45.5
	130–139 mmHg	4	18.2
	≥140 mmHg	3	13.6
Diastolic blood pressure	<80 mmHg	9	40.9
	80–89 mmHg	7	31.8
	≥90 mmHg	6	27.3
Pulse	60–100 beats/minute, normal	16	72.7
	>100 beats/minute	6	27.3
Respiratory rate	>16 breaths/minute	22	100.0
BMI category, kg/m²	Normal	12	54.5
	Pre-obese	6	27.3
	Obesity class I	2	9.1
	Obesity class II	2	9.1

Medical comorbidity was documented in 10 patients (45.5%). Fourteen patients (63.6%) had at least one previous admission. Psychiatric, medical, and family-history variables were incompletely documented for some patients and are summarized using available documentation (Table 6).

**Table 6:** Psychiatric, medical, and family history (N=22)

Psychiatric, Medical, and Family History		Frequency		Percentage
Previous admission	None	8	36.4	
	1	3	13.6	
	2	2	9.1	
	3 or more	9	40.9	
Family history of mental illness?	No	2		100.0
Family history of substance use?	Yes	4		80.0
	No	1		20.0
Comorbid Mental Disorders?	Yes	1		5.9
	No	16		94.1
on psychotropic medications?	Yes	5		22.7
	No	17		77.3
If on psychotropic meds, is patient complaint? N=3	Yes	3		100.0
Comorbid medical condition?	Yes	10		45.5
	No	12		54.5
On regular health medications?	Yes	5		23.8
	No	16		76.2
If yes, compliant on medications?	Yes	1		33.3
	No	2		66.7

Treatment approaches

Buprenorphine-based OAT was the main pharmacological approach. The most common treatment pathway was buprenorphine/naloxone sublingual therapy followed by weekly injectable buprenorphine, used in 12 patients (54.5%). Seven patients (31.8%) transitioned from weekly to monthly injectable buprenorphine. Two patients (9.1%) received supportive treatment, and one (4.5%) received buprenorphine/naloxone sublingual therapy only (Table 7).

**Table 7:** Treatment regimens, discharge outcomes, and follow-up data (N=22) DAMA: discharged against medical advice; PO: *per os* (orally); HS: *hora somni* (at bedtime)

Discharge Outcome and Follow-Up		Frequency	Percentage
Type of treatment received	Supportive treatment	2	9.1
	Only buprenorphine/naloxone tablets	1	4.5
	Received buprenorphine/naloxone tabs and switched to buprenorphine weekly injection	12	54.5
	Switched from buprenorphine weekly injections to buprenorphine monthly injection	7	31.8
Discharge outcome	Normal discharge to home with OPD appointment	5	22.7
	Normal discharge to rehab services (mid-way house, day care)	9	40.9
	DAMA	8	36.4
Discharge condition	Stable	22	100.0
Discharged on Home medication?	Yes	18	81.8
	No	4	18.2
If yes, what are the medications and doses? (n=18)	Buprenorphine 16 mg weekly injection	7	38.9
	Buprenorphine 24 mg weekly injection	1	5.6
	Buprenorphine 64 mg monthly injection	4	22.2
	Buprenorphine 96 mg monthly injection	4	22.2
	Psychotropics: mirtazapine 15 mg PO HS, risperidone 2 mg HS syrup, desvenlafaxine 50 mg PO, olanzapine 10 PO HS, Depaken 500 mg PO HS	2	11.1
Discharge Diagnosis (If not available, write NA)	Opioid use disorder	16	72.7
	Polysubstance use disorder	6	27.3
Lost follow-up within the last 6 months after discharge	Yes	5	22.7
	No	17	77.3
Readmission within 6 months of discharge	Yes	6	27.3
	No	16	72.7
Duration between last discharge and readmission	≤6 months	3	50
	>6 months	3	50
If there is a urine toxicology test within 6 months of discharge, is it positive for opioids?	Yes (+ve)	5	33.3
	No	10	66.7
If there is a urine toxicology test within 6 months of discharge, is it positive for substances other than opioid?	Yes (+ve)	5	33.3
	No	10	66.7
If yes, what is/are the substance?	Amphetamine	2	40.0
	Benzodiazepine	1	20.0
	Amphetamine/Cannabis	1	20.0
	Amphetamine/Benzodiazepine/Cannabis	1	20.0

Adjunctive medications were used for symptom control when clinically indicated, including non-opioid analgesics, sedative-hypnotics for insomnia, and antiemetics for nausea. Psychosocial interventions were initiated during admission, although intensity varied according to length of stay and patient engagement.

Discharge outcomes

Normal discharge occurred in 14 patients (63.6%): five (22.7%) were discharged home with an outpatient department appointment, and nine (40.9%) were discharged to rehabilitation services. Discharge against medical advice (DAMA) occurred in eight patients (36.4%). All patients (n = 22, 100.0%) were clinically stable at discharge.

During follow-up, six patients (27.3%) were readmitted within six months, and five (22.7%) were lost to follow-up. Urine toxicology testing within six months was available for 15 patients (68.2%); among those tested, five (33.3%) had a positive opioid screen and five (33.3%) had a positive screen for a non-opioid substance (Table 7).

An exploratory association was observed between treatment type and discharge outcome (p = 0.005). Because the sample was small and several cells were sparse, this result should be interpreted cautiously and should not be considered evidence of causality or treatment effectiveness (Table 8).

**Table 8:** Association between treatment type and discharge/follow-up outcomes (N=22) ^a^ Significant at <0.05; using Fisher's Exact Test DAMA: discharged against medical advice; PO: *per os* (orally); HS:* hora somni *(at bedtime)

Discharge outcome and Follow-Up		Total	Type of treatment received				P-value
			Supportive treatment, n (%)	Only buprenorphine/naloxone tablets, n (%)	Received buprenorphine/naloxone tabs and switched to buprenorphine weekly injection, n (%)	Switched from buprenorphine weekly injections to buprenorphine monthly injection, n (%)	
Discharge outcome	Planned discharge to home with OPD appointment	5	2 (40.0%)	0 (0.0%)	2 (40.0%)	1 (20.0%)	0.005^a^
	Planned discharge to rehab services (mid-way house, day care)	9	0 (0.0%)	0 (0.0%)	3 (33.3%)	6 (66.7%)	
	DAMA	8	0 (0.0%)	1 (12.5%)	7 (87.5%)	0 (0.0%)	
Discharge condition	Stable	22	2 (9.1%)	1 (4.5%)	12 (54.5%)	7 (31.8%)	-
Discharged on home medication?	Yes	18	2 (11.1%)	0 (0.0%)	9 (50.0%)	7 (38.9%)	0.122
	No	4	0 (0.0%)	1 (25.0%)	3 (75.0%)	0 (0.0%)	
If yes, what are the medications and doses?	Buprenorphine 16 mg weekly injection	7	0 (0.0%)	0 (0.0%)	7 (100.0%)	0 (0.0%)	<0.001^a^
	Buprenorphine 24 mg weekly injection	1	0 (0.0%)	0 (0.0%)	1 (100.0%)	0 (0.0%)	
	Buprenorphine 64 mg monthly injection	4	0 (0.0%)	0 (0.0%)	1 (25.0%)	3 (75.0%)	
	Buprenorphine 96 mg monthly injection	4	0 (0.0%)	0 (0.0%)	0 (0.0%)	4 (100.0%)	
	Psychotropics: Mirtazapine 15 mg PO HS, risperidone 2 mg HS syrup, desvenlafaxine 50 mg PO	2	2 (100.0%)	0 (0.0%)	0 (0.0%)	0 (0.0%)	
Discharge Diagnosis	Opioid use disorder	16	1 (6.3%)	1 (6.3%)	8 (50.0%)	6 (37.5%)	0.634
	Polysubstance use disorder	6	1 (16.7%)	0 (0.0%)	4 (66.7%)	1 (16.7%)	
Lost to follow-up within the last 6 months after discharge	Yes	5	1 (20.0%)	0 (0.0%)	4 (80.0%)	0 (0.0%)	0.272
	No	17	1 (5.9%)	1 (5.9%)	8 (47.1%)	7 (41.2%)	
Readmission within 6 months of discharge	Yes	6	0 (0.0%)	1 (16.7%)	3 (50.0%)	2 (33.3%)	0.466
	No	16	2 (12.5%)	0 (0.0%)	9 (56.3%)	5 (31.3%)	
Duration between last discharge and readmission	≤6 months	3	0 (0.0%)	1 (33.3%)	1 (33.3%)	1 (33.3%)	0.657
	>6 months	3	0 (0.0%)	0 (0.0%)	3 (100%)	1 (25.0%)	
If there is a urine toxicology test within 6 months of discharge, is it positive for opioids?	Yes (+ve)	5	0 (0.0%)	0 (0.0%)	4 (80.0%)	1 (20.0%)	0.580
	No	10	0 (0.0%)	0 (0.0%)	5 (50.0%)	5 (50.0%)	
If there is a urine toxicology test within 6 months of discharge, is it positive for substances other than opioid?	Yes (+ve)	5	0 (0.0%)	0 (0.0%)	4 (80.0%)	1 (20.0%)	0.580
	No	10	0 (0.0%)	0 (0.0%)	5 (50.0%)	5 (50.0%)	

## Discussion

This cross-sectional study describes the demographic, clinical, substance-use, treatment, discharge, and early follow-up characteristics of male inpatients with confirmed OUD treated at a specialized addiction facility in Riyadh. The findings provide setting-specific insight into inpatient OUD care after the introduction of buprenorphine-based OAT, but they should not be interpreted as national estimates or as evidence of treatment effectiveness because of the small, single-center design.

Patient characteristics

Participants had a mean age of 38.3 years, indicating that most patients presented for inpatient treatment during adulthood. This pattern is broadly consistent with Saudi treatment-center and hospital-based studies showing that individuals with substance use disorders commonly reach formal treatment after several years of substance use [5-9]. In the present cohort, non-employment (86.4%), single marital status (63.6%), and documented legal issues (45.5%) were common. Similar social vulnerabilities have been described in Saudi clinical and treatment-seeking populations and may affect treatment engagement, discharge planning, and continuity of care [5-9].

Heroin was the most frequently documented opioid in this cohort (63.6%), followed by morphine sulfate (27.3%). This finding is compatible with previous Saudi treatment-center literature describing heroin use among selected clinical samples [5-7]; however, such data should not be generalized to national substance-use patterns. Polysubstance use was documented in 90.9% of patients, most commonly involving methamphetamine/amphetamine and cannabis. This is clinically important because comorbid non-opioid substance use disorders are common among individuals with OUD internationally and may complicate withdrawal management, treatment engagement, and relapse prevention [4,5,8].

Clinical presentation and comorbidities

Most patients had used opioids recently before admission, with 59.1% reporting last opioid use within 24 hours. Withdrawal symptoms were documented in 68.2% of patients; among those with documented symptoms, disturbed sleep or insomnia, muscle or body aches, and sweating were most frequent. These findings are consistent with the expected clinical presentation of OUD and opioid withdrawal in inpatient settings [1,2]. Two patients (9.1%) required high-dependency, medical care, or intensive care monitoring, emphasizing the need for close medical assessment during withdrawal management, particularly in patients with injection use, polysubstance use, or medical comorbidity.

Bloodborne viral infections were frequent in this cohort. HCV seropositivity was documented in 66.7%, while HIV and HBV were each documented in 9.5%. These findings are comparable to a retrospective study of male Saudi inpatient heroin injectors in the central region of Saudi Arabia, which reported HCV antibody seropositivity in 77.8%, HIV infection in 9.8%, and HBV surface antigen positivity in 7.7% [10]. However, both the current findings and prior Saudi reports describe selected treatment-seeking patients or inpatient heroin injectors populations and should not be interpreted as national prevalence estimates. The high burden of infectious disease supports comprehensive infectious disease assessment and care within addiction services, including screening, confirmatory testing, specialist referral, monitoring, treatment when indicated, behavioral counseling, and psychosocial support. Psychiatric and medical comorbidities were also relevant, although psychiatric diagnoses were not consistently documented in the medical records. This limitation is important because psychiatric comorbidity and polysubstance use are frequently reported among people with substance use disorders and may influence treatment adherence, relapse risk, and post-discharge outcomes [4,5,8].

Treatment patterns

Buprenorphine-based OAT was the main pharmacological strategy in this cohort. Twelve patients (54.5%) received buprenorphine/naloxone sublingual therapy followed by transition to weekly injectable buprenorphine, and seven patients (31.8%) transitioned from weekly to monthly injectable buprenorphine. Buprenorphine-based treatment is an established pharmacological approach for OUD and may support withdrawal management, craving reduction, and continuity of care when combined with psychosocial and follow-up services [11-13].

An exploratory association was observed between treatment type and discharge outcome. However, this finding must be interpreted cautiously because only 22 patients were included, and several treatment and discharge categories had sparse cell counts. Therefore, the association should be considered hypothesis-generating and should not be interpreted as causal, as evidence of an independent treatment effect, or as the comparative effectiveness of one formulation over another. Larger multicenter studies with standardized clinical outcomes and follow-up are required before firm conclusions can be drawn.

Post-discharge outcomes

Post-discharge outcomes showed ongoing vulnerability after inpatient care. Within six months, 27.3% of patients were readmitted, and 22.7% were lost to follow-up. Readmission after substance-use treatment has been reported in Saudi rehabilitation settings and reflects the chronic, relapsing nature of OUD and the difficulty of maintaining engagement after discharge [1,2,9]. Among patients with available urine toxicology testing, 33.3% were opioid-positive, and 33.3% were positive for non-opioid substances. Because toxicology testing was available for only 15 patients, these results should be interpreted cautiously and may underestimate post-discharge substance use. The high prevalence of polysubstance use, legal issues, and social instability in this cohort suggests that discharge planning should extend beyond withdrawal stabilization. Linkage to outpatient follow-up, rehabilitation services, psychosocial support, and case management may be particularly important for patients with multiple relapse vulnerabilities [4,5,9,11].

Implications for clinical practice and policy

Several practice implications arise from these findings. First, infectious disease services should be integrated into addiction care as comprehensive assessment and management rather than medication provision alone, particularly for patients with injection opioid use [10]. Second, buprenorphine-based OAT, including long-acting injectable formulations, may support continuity of care for selected patients when embedded within structured psychosocial and follow-up pathways [11-13]. Third, patients with polysubstance use, legal problems, non-employment, or limited social support may require early discharge planning and active linkage from inpatient care to outpatient, rehabilitation, and community-based services [5,9,11].

Limitations

This study has important limitations. The small sample size and single-center design limit generalizability, and the exclusively male cohort does not represent the full spectrum of OUD presentations in Saudi Arabia. The retrospective design depended on medical record documentation, which was incomplete for several variables, including psychiatric comorbidity, craving, adverse events, and post-discharge toxicology. Follow-up urine toxicology was not uniformly available, which may have led to underestimation of relapse or continued substance use. The exploratory association analysis was limited by sparse cell counts and should not be used to infer causality or treatment effectiveness. Larger multicenter studies with standardized assessment, inclusion of female patients, and structured post-discharge follow-up are needed to strengthen the evidence base for OUD treatment in Saudi Arabia.

## Conclusions

This study provides updated insight into the demographic, clinical, and treatment characteristics of male inpatients with OUD in Riyadh, Saudi Arabia. The findings highlight a high prevalence of polysubstance use, medical comorbidities, and social vulnerabilities, including non-employment and legal issues, underscoring the complexity of managing OUD in this population. The observed association between treatment type and discharge outcome suggests that structured buprenorphine-based regimens, including long-acting injectable formulations, may support planned discharge and facilitate continuity of care, yet it is preliminary and requires confirmation in larger studies.

Post-discharge challenges remained evident, including readmission and loss to follow-up within six months. These findings emphasize the need for integrated inpatient-to-community treatment pathways, including rehabilitation linkage, harm reduction strategies, infectious disease care, and comprehensive psychosocial support. Expanding access to long-acting buprenorphine formulations beyond specialized centers, combined with targeted interventions for high-risk groups such as polysubstance users and those with legal problems, could enhance treatment retention and outcomes in Saudi Arabia.
